# Glass–Ceramics in Dentistry: A Review

**DOI:** 10.3390/ma13051049

**Published:** 2020-02-26

**Authors:** Le Fu, Håkan Engqvist, Wei Xia

**Affiliations:** 1 School of Materials Science and Engineering, Central South University, Changsha 410083, China; 2 Applied Materials Science, Department of Engineering Science, Uppsala University, 751 21 Uppsala, Sweden; Hakan.Engqvist@angstrom.uu.se

**Keywords:** glass–ceramics, dental prostheses, strength, translucency, strengthening mechanisms

## Abstract

In this review, we first briefly introduce the general knowledge of glass–ceramics, including the discovery and development, the application, the microstructure, and the manufacturing of glass–ceramics. Second, the review presents a detailed description of glass–ceramics in dentistry. In this part, the history, property requirements, and manufacturing techniques of dental glass–ceramics are reviewed. The review provided a brief description of the most prevalent clinically used examples of dental glass–ceramics, namely, mica, leucite, and lithium disilicate glass–ceramics. In addition, we also introduce the newly developed ZrO_2_–SiO_2_ nanocrystalline glass–ceramics that show great potential as a new generation of dental glass–ceramics. Traditional strengthening mechanisms of glass–ceramics, including interlocking, ZrO_2_–reinforced, and thermal residual stress effects, are discussed. Finally, a perspective and outlook for future directions in developing new dental glass–ceramics is provided to offer inspiration to the dental materials community.

## 1. The History of Glass–Ceramics and Dental Glass–Ceramics

Synthetic glass–ceramics were serendipitously discovered by Stanley Donald Stookey in 1953. [1,2,3,4]. After the discovery of lithium disilicate glass–ceramic, Corning Inc. developed and commercialized two new glass–ceramics based on Li–aluminosilicates (LAS) and Mg–aluminosilicates (MAS) during 1953–1963 [5]. The LAS glass–ceramic was used as cookware because of its very low coefficient of thermal expansion (CTE). The development of MAS glass–ceramic was motivated by the need arose for a ceramic missile nosecone for a missile to be guided by an internal antenna [1]. Between 1963 and 1980, researchers tried to develop transparent and nano–crystalline glass–ceramics. For instance, nano–crystalline β–quartz glass–ceramic introduced by Schott has a crystalline size of about 50 nm [6].

**Figure 1:**
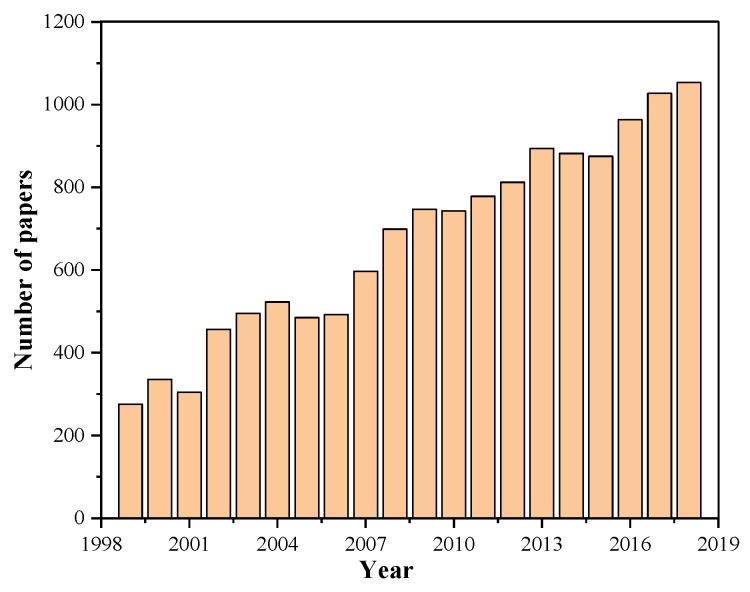
An idea of scientific and commercial significance of glass–ceramics. The number of published papers searched from Web of Science with the key words “glass–ceramics”.

In the last two decades, glass–ceramics have attracted great interests of people in scientific community. Figure 1 provides an idea of the scientific significance of glass–ceramics in terms of published papers. There are only 276 papers in 1999, however, the number keeps increasing over the last 20 years, reaching to approximately 1100 in 2018 (Figure 1). This indicates that more and more material scientists in research institutes and universities become interested in glass–ceramics.

Humans have long been aware of the medical and esthetic benefits of tooth replacements. Ancient Egyptians produced esthetic tooth replacements using bovine teeth. Ceramic materials for dental restorations were first invented in the 18th century [7]. Aesthetics (adequate translucency) and durability (adequate strength and chemical stability) are the two attributes of ceramics over other materials in terms of being used as dental materials.

In 1962, the first two US patents porcelain–fused–to–metal (PFM) restorations were awarded which consisted of gold alloy and feldspathic porcelain [8]. Since then PFM restorations have set the standard for multiple teeth restoration. In the past decades, dental bridges were mostly metal–porcelain composite, consisting of a metallic framework for load–bearing and coated porcelain for aesthetic appearance [9]. Despite the wide application of PFM restorations, the use of metals in the oral cavity has come under disputes in recent years because of their biological incompatibility and some other concerns, such as chipping of the veneering layer because of the CTE differences between porcelain veneering layer and metallic framework [4,5]. These are the main factors motivating continued research into all–ceramic restorations. All–ceramic dental restorations are available on the market since the 1980s. Yttrium–stabilized tetragonal ZrO_2_ (Y–TZP) has gained remarkable popularity in dentistry because of its excellent mechanical properties. However, Y–TZP has low translucency. Thus, it still requires a veneering layer constructed with a compatible porcelain in order to achieve a more favorable aesthetic result [10]. This is so–called multilayered dental prostheses. However, the problem of chipping of the veneering layer still exists in the multilayered restorations [10]. This drives the development of monolithic prostheses with high strength and high translucency in recent years.

Dental glass–ceramics are highly attractive for dentists and patients owing to their combination of excellent physical and chemical properties, such as outstanding esthetics, translucency, low thermal conductivity, adequate strength, biocompatibility, wear resistance, and chemical durability [11,12]. In 1984, Corning Inc. was the first company to fabricate glass–ceramic material for dental restorations [4,9]. The attempts of developing glass–ceramics with higher strength through chemical composition modification and optimization of the manufacturing process have never ended. Dispersion strengthening is one of the well–grounded approaches to strengthening glass–ceramic. One of the most successful particle fillers used in dental glass–ceramics is leucite. One example of commercial dental ceramics containing leucite as a strengthening phase shows a bending strength of ~138 MPa (Ivoclar Vivadent, Liechtenstein) [13]. Currently, the most widely used, the strongest and toughest dental glass–ceramics are made with lithium disilicate. The glass–ceramic contains ~70 vol% of interlocked rod–like lithium disilicate crystals. The material possesses a flexural strength of 350 MPa and a fracture toughness of 2.9 MPa m^1/2^ [14,15], which were more than twice those of leucite–based glass–ceramics.

This paper reviews some aspects of the field, including microstructure and preparation of glass–ceramics, manufacturing of dental glass–ceramics, commercial and newly–developed dental glass–ceramics, strengthening mechanisms, and also our perspective for future directions.

## 2. Properties and Applications of Glass–Ceramics

**Figure 2:**
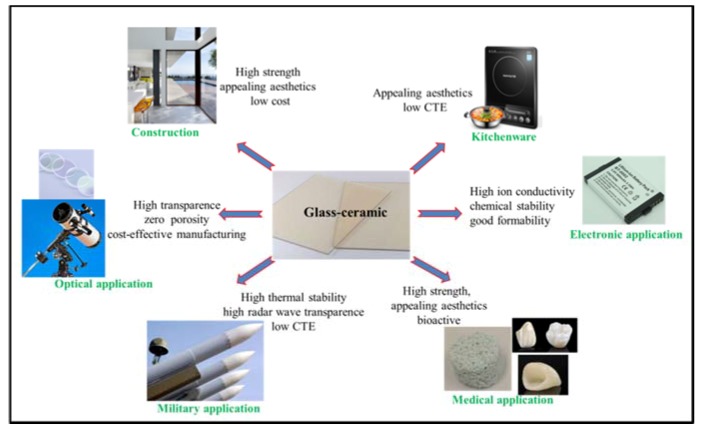
Applications of glass–ceramics in a wide range of fields.

Glass–ceramics have been widely used in a wide range of fields in our daily life, owning to their challenging combination of properties to fulfil specific requirements. Figure 2 demonstrates some of the applications of glass–ceramics in many fields. In construction field, one of the most popular glass–ceramic used in construction is Neopariés LT, with wollastonite as the main crystalline phase [1]. Neopariés glass–ceramic panels are an ideal alternative to stone for interior and exterior applications. In optical field, many glass–ceramics show high translucency or even can be transparent because of the fact that zero porosity can be relatively easily achieved [16,17,18]. These make glass–ceramics excellent material for optical applications. For instance, transparent and low thermal expansion glass–ceramics based on lithium aluminosilicate (LAS) system have been used as telescope mirror blanks and laser gyroscopes [18]. In military field, glass–ceramics now are used in nosecones of high–performance aircraft and missiles. Materials used in these applications must exhibit a challenging combination of properties to withstand critical conditions resulting from high–speed flying in the atmosphere: Low coefficient of thermal expansion; high mechanical strength; high abrasion resistance; high radar wave transparency for navigation [1,6]. In medical field, bioglass has been successfully used in the medical field [12,19]. However, the inherent low strength and low toughness limit the application of bioglass as a load–bearing biomaterial. With crystalline phases as strengthening and toughening phases, glass–ceramics overcome the weakness of bioglass. For instance, A–W glass–ceramic that contains apatite and β–wollastonite (CaO·SiO_2_) crystals (with the commercial brand name of Cerabone) is considered as the most outstanding bioactive glass–ceramics for hard tissue repair [3]. In electronic field, all–solid–state secondary batteries with inorganic solid electrolytes are expected to be next–generation high–output batteries. Different types of inorganic solid electrolytes made by glass–ceramics have been developed, for instance, Inda et al. [20] showed that glass–ceramics has the crystalline form of Li_1+x+y_Al_x_Ti_2−x_Si_y_P_3−y_O_12_ exhibited a high lithium–ion conductivity of 10^−3^ S·cm^−1^. In kitchenware field, higher toughness (compared with glass), appealing aesthetics, and very low thermal expansion coefficient make glass–ceramics the excellent material for kitchenware, such as cooktops, cookware, and bakeware. The most widely used system is the Li_2_O–Al_2_O_3_–SiO_2_ (LAS) system with additional components, such as CaO, MgO, ZnO, etc., [1,6]. The main crystalline phase is a β–quartz solid solution, which has an overall negative CTE. LAS glass–ceramics can sustain repeated and quick temperature changes of 800 to 1000 °C [3,21].

## 3. Microstructure and Preparation of Glass–Ceramic

### 3.1. Microstructure Differences between Glass, Glass–Ceramic, and Ceramic

**Figure 3:**
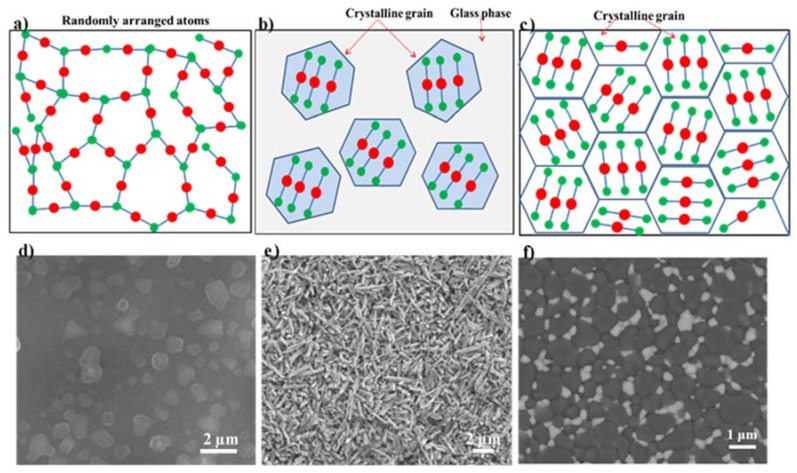
Microstructure differences between glass, glass ceramic, and ceramic: schematic microstructures of glass (**a**), glass–ceramics (**b**), and ceramic (**c**). Corresponding examples of glass (**d**), glass–ceramics (**e**), and ceramic (**f**). SEM image of non–annealed Li_2_O–SiO_2_ glass. Reprinted from ref [22] with permission. (**e**) SEM images of lithium disilicate glass–ceramic after etching. (**f**) SEM image of zirconia toughened alumina ceramics, with ZrO_2_ showing light contrast and Al_2_O_3_ showing dark contrast. Reprinted from ref [23] with permission.

Figure 3 describes the structure differences between glass, glass–ceramics, and ceramic. Strictly speaking, the term “glass” describes a state of matter where the atoms/molecules are randomly arranged, in other words, glass materials are amorphous (Figure 3a). Figure 3d shows an example of a glass in a Li_2_O–SiO_2_ system, in which droplets with slightly brighter contrast were embedded in the glass matrix with darker contrast [22]. Metastable immiscibility that occurs in binary Li_2_O–SiO_2_ system causes segregation of the glass phase into droplet–like zones of Li–rich phase and SiO_2_–rich glass matrix [22]. Ceramic materials are mainly composed of crystalline grains, with a small amount of glass phase at grain boundaries (Figure 3c). Figure 3f reveals the microstructure of zirconia toughened alumina ceramic; it can be seen that ZrO_2_ grains (light contrast) and Al_2_O_3_ grains (dark contrast) are connected to each other with grain boundaries [23]. Glass–ceramics are a special group of material consisting of at least one crystalline phase and glassy matrix (Figure 3b). Crystalline phase(s) are embedded in the glass matrix. The crystallinity varies most frequently between 30 and 70%. Two types of interfaces can be found in glass–ceramics; one is the interface between crystalline phases, and the other is the interface between the crystalline phase and the glassy matrix. Figure 3e demonstrates the microstructure of an LD glass–ceramic. Glass phase has been removed by acid etching, leaving rod–like Li_2_Si_2_O_5_ crystalline phase with a length of 3 to 6 µm.

### 3.2. Preparation of Glass–Ceramic

**Figure 4:**
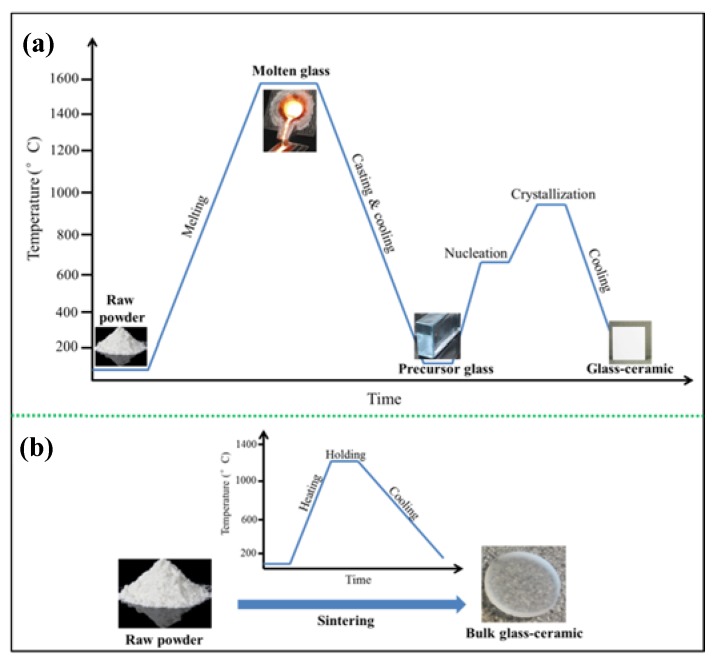
The two manufacturing processes of glass–ceramics: (**a**) The classic melting–casting–annealing process; (**b**) the concurrent sinter–crystallization process.

There are two ways to prepare glass–ceramics. Classically, a glass–ceramic is made through controlled heat treatment of a precursor glass, known as ceramming. Glass–ceramics also can be produced by concurrent sintering–crystallization of glass–particle compacts. The manufacturing of glass–ceramic using classic melting–casting–annealing processes involves three general steps [4] (Figure 4a).

First, preparation of raw materials, glass–forming components, and nucleating agents are mixed with ball milling [24]. The nucleating agents are used to stimulate nucleation in the following annealing process; Second, the batch is melted and then cooled to room temperature to form a precursor glass. A homogeneous molten glass is formed by heating the raw materials to elevated temperature in a high–temperature furnace. The melt is then casted into a mold with the desired shape. After cooling to room temperature, a precursor glass forms.

Third, the precursor glass is then annealed to induce crystallization, thereby forming glass–ceramic. This process is known as ceramming [25]. The formation of crystalline phases in glass–ceramics comprises two main steps. In the first step, the precursor glass is heated to a temperature slightly above the transformation range and maintained for a sufficient time to achieve substantial nucleation. The addition of nucleating agents results in volume or bulk nucleation. Homogeneously dispersed nano–crystals precipitate from the glass matrix [17]. Different nucleation agents are needed for different glass–ceramic systems. For instance, the most frequently used nucleating agents for the Li_2_O–Al_2_O_3_–SiO_2_ system are ZrO_2_, TiO_2_, or both [17,26]. In the second step, the nucleated body is heated to a higher temperature to allow the growth of crystals on these nuclei. Types of nucleation agent and thermal treatments during nucleation and crystallization processes are two of the most critical factors that determine the final microstructure of glass–ceramics. A wide range of microstructures can be created, including uniform crystal phases [17], inter–locking crystals [27], and crystals with a wide variety of shapes and sizes [28,29].

**Figure 5:**
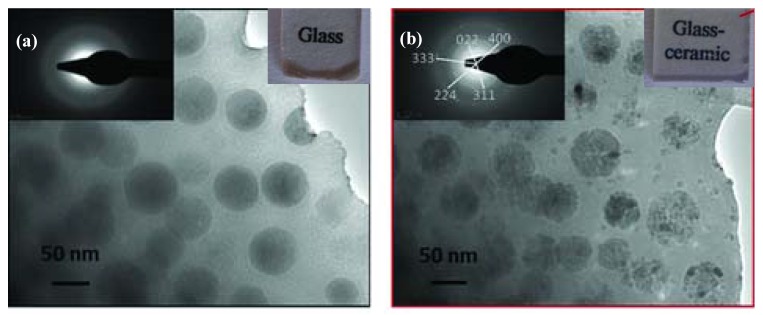
An example demonstrating the microstructure evolution during the ceramming process. TEM micrograph of an 80 GeO_2_–10ZnO–10Ga_2_O_3_ (+2.5 Na_2_O) (mol%) glass with phase separation (**a**) and corresponding glass–ceramic after ceramming (**b**). Reprinted from ref [30] with permission.

Figure 5 demonstrates the microstructure evolution during ceramming [30]. The precursor glass materials exhibit nanoscale phase separation, with spherical droplets (dark contrast) distributed homogeneously in the matrix (bright contrast) (Figure 5a). The inserted selected area electron diffraction (SAED) patterns present a halo pattern, indicating that the material is amorphous. During ceramming, nanoscale crystals form and grow in the droplet glass (Figure 5b). The inserted SAED patterns reveal a polycrystalline structure. Thus, the precursor glass becomes glass–ceramic after ceramming (Figure 5).

Figure 4b schematically reveals the process of manufacturing glass–ceramic through concurrent sintering–crystallization of glass–particle compacts. Like the above melting–casting–annealing process, the first step of the concurrent sinter–crystallization process is the preparation of raw powder. There are several ways to prepare raw powder, either by directly mixing oxides [31], or by the melting–quenching method to form cullet [32], or by the sol–gel method [33,34]. Crystallization occurs during the sintering process. Compared with the melting–casting–annealing process, the main advantage of the sinter–crystallization process is that nucleating agents are not needed. Moreover, there are fewer steps in the sinter–crystallization process.

## 4. Property Requirements of Dental Prostheses

Teeth primarily consist of enamel, dentine, and pulp. If lost or damaged, a tooth cannot be repaired or regenerated. Restorative dentistry is concerned with the repair of damaged teeth and their supporting structures. Basically, there are three property requirements for a material intended to be used as dental prostheses: mechanical strength, esthetics, and chemical solubility.

### 4.1. Mechanical Properties

Mechanical properties are one of the most important properties of dental prostheses since they act as a load–bearing biomaterial. The stress distribution of dental prostheses is complex, largely dependent on the geometry of the dental prostheses [35]. Strength is one of the most important criteria for dental prostheses. Average chewing forces during normal mastication are reported in a wide range from 40 to 440 N [36] Higher forces can readily be reached for brief periods (~500 to ~880 N) [36]. For dental glass–ceramics, although occlusal loading is nominally compressive, some tensile stresses in individual “dome–like” crown or in frameworks with connectors are developed at some sites. Cracks tend to follow paths where these tensile stresses are greatest [35]. Fracture toughness is a vital factor that determines the quality of a dental glass–ceramic, since glass–ceramic is a brittle material [37].

Another important mechanical characteristic for the long–term success of a restoration is microhardness. On one hand, glass–ceramics with high hardness show less wear at the top surface of dental prostheses, therefore suppressing contact damage. Thus, high hardness is beneficial to dental prostheses. Whereas, on the other hand, high hardness of dental prostheses results in high wear rate of the antagonist enamel during chewing. Thus, a balance needs to be maintained between the above two aspects. The Vickers hardness of human dental enamel is approximately 400 [12]. It is better to match the hardness of dental prostheses to human dental enamel to reduce the wear the antagonist enamel.

### 4.2. Esthetics

The esthetics of dental ceramics are characterized by two optical properties, namely: color and translucency. Certain translucency or opacity of dental glass–ceramics is needed according to their intended clinical use. For PFM, the color of the metal framework needs to be masked by an opaque layer before the more translucent and more esthetic layers are laid down [4,38]. Glass–ceramics with higher opacity have greater hiding power. Thus, a thinner layer of opaque glass–ceramic is needed, which leaves more room for the more translucent esthetic ceramic layers [4]. Whereas, for monolithic glass–ceramic prostheses, certain translucency is necessary to mimic the optical properties of natural teeth of different patients.

### 4.3. Chemical Resistance

Dental glass–ceramics are biomaterials that need to stay in the human oral cavity body for a long time (more than 10 years). Thus, for glass–ceramics to survive not only do they need to be strong and tough enough to resist the biting forces (as discussed above), they also have to be able to resist the acidic/alkaline corrosive environment in the oral cavity at approximately 37 °C [39]. According to international standard ISO 6872 [40], dental glass–ceramics intended for different clinic uses have different chemical solubility requirements. For instance, the chemical solubility of monolithic ceramic for single–unit anterior prostheses, veneers, inlays, or onlays must be less than 100 μg/cm^3^ [40]. In comparison, partially or fully covered substructure ceramic for single–unit anterior or posterior prostheses should have a chemical solubility of less than 2000 μg/cm^3^ [40].

## 5. Manufacturing of Dental Restorations

Dental restorations can be fabricated by different methods: powder condensation (conventional powder slurry ceramics/glass–ceramics), lost–wax/heat pressed technique (pressable ceramics/glass–ceramics), slip casting (infiltrated ceramics), and CAD/CAM (computer–aided design and computer–aided manufacturing) technique (machinable ceramics/glass–ceramics) [41]. The CAD/CAM technique is selected to be discussed in detail since the technique is currently the most widely used manufacturing technique. Additive manufacturing (AM), as a developing and promising technique, has received much attention in dentistry. This is a future–oriented technique. Thus, what has been achieved so far and problems need to be solved in the future related to AM in dentistry will also be discussed.

### 5.1. CAD–CAM Workflow

**Figure 6:**
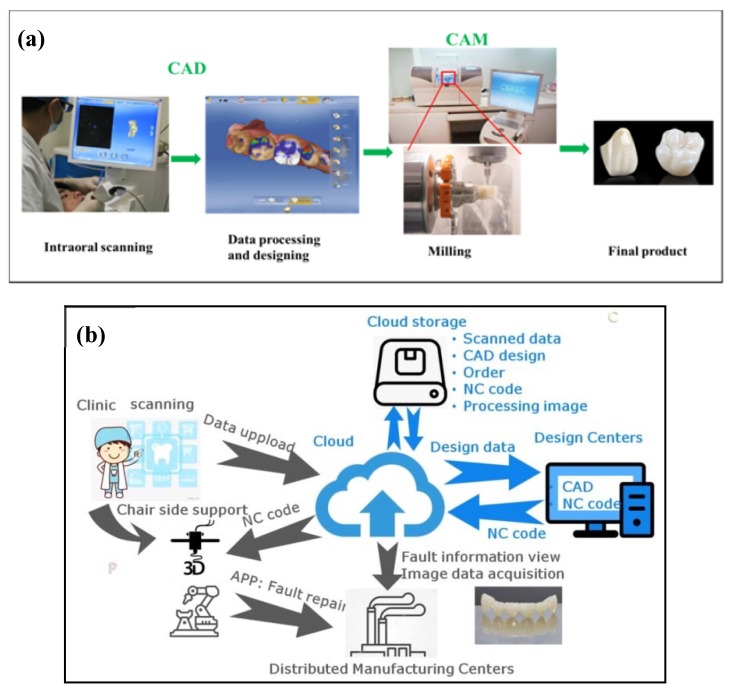
Computer–aided design and computer–aided manufacturing (CAD–CAM)–based workflow in dentistry. (**a**) The Cerec workflow includes three steps: first, a intraoral canner is used to acquire optical images of the prepared teeth; second, raw scanning data is processed with the aid of the chairside software, followed by the design of the restoration; third, CAM technology takes the designed model to a computer numeric control machine to manufacture the restoration. (**b**) The novel cloud connected digital dentistry system. The full worldwide digital platform is characterized by the separation of design work to form independent design centers from the convention production centers. Reprinted from [45].

Figure 6a shows the CAD–CAM workflow. First, optical images of the prepared teeth are obtained through intraoral scanning. CAD technology uses software to define the shape and dimensions of the restoration; Second, CAM technology takes the designed model to manufacture the restoration with a micro milling machine, usually from a block made of dental material. The last step is to bond/cement the newly prepared restoration to the surface of the prepared natural tooth, in which adaptation plays an important role in the success of any restoration. Poor marginal adaptation may cause many problems, such as plaque accumulation, periodontal disease, and endodontic inflammation [42,43].

In the CAM step, there are two types of milling. The first one is the machining of the prosthetic restoration from a block of the sintered material, which is known as “hard milling,” the second one is the machining of a block in a partially sintered state, followed with a subsequent final sintering step in a furnace, which is known as “soft milling.” Hard milling with CAD/CAM technique provides the restoration with greater precision of its contours and shape. The introduction of hard milling with CAD/CAM technology to restorative dentistry allows the production of dental frameworks made of zirconia with high accuracy (e.g., DC–Zirkon/DCS Dental AG, Denzir/Cadesthetics AB). IPS e.max ZirCAD developed by Ivoclar Vivadent is a fully sintered ZrO_2_–based all–ceramic restorations that are manufactured by hard milling [44]. However, one of the drawbacks if machining of fully sintered and strong ceramic blocks is heavy abrasion of milling tools. Soft milling has been widely used to manufacture dental prosthesis made of lithium disilicate glass–ceramic. This is discussed in detail in the following section.

The new trend of digital dentistry workflow is to separate designing from manufacturing. The skills and expertise among dentists, dental engineers/technicians, software developers, and materials manufactures can be integrated into a modern system. A common source of digital data can be communicated between dentists, dental engineers/technicians, and manufactures through long distance with Internet. The new full digital workflow is known as Completely Digital Design and Completely Digital Manufacture (CDD/CDM) (Figure 6b) [45]. Through strengthening the collaboration among clinics, labs, design and manufacture centers, this new workflow would also gain improved efficiency/accuracy/reliability, as well as the predictable and visualized results for meeting the patient satisfaction. More detail information about the novel cloud connected dental system can be found in ref [45].

### 5.2. Additive Manufacturing (AM) Technique

**Figure 7:**
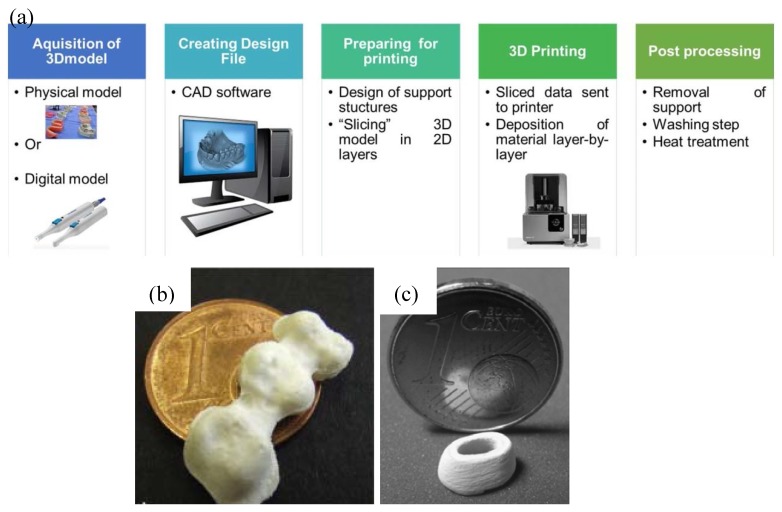
Additive manufacturing in dentistry. (**a**) The process of manufacturing a dental prosthesis through additive manufacturing. Reprinted with permission of Ref [47]. (**b**) Zirconia framework prepared by direct inkjet printing (DIP) technology. Reprinted with permission of Ref [51]. (**c**) A tooth model consisting of 35 layers printed by SLM technology. The composition is 25.5Al_2_O_3_–74.5SiO_2_ (wt%). Reprinted with permission of Ref [52].

Although the CAD–CAM technique has already been well established in dentistry [46], the major drawback of this technique is the great waste of material upon machining since it is a subtractive manufacturing method. The waste corresponds to approximately 90% of the prefabricated block in some cases and leftovers from these are not reusable. AM technique, also known as 3D printing, could be an effective new technology to overcome this problem. Meanwhile, the rising demand for custom–tailored and patient specific dental products renders dentistry to be one of the rapidly expanding segments of AM [47]. AM involves processing methodologies that are capable of producing structures by depositing materials layer–by–layer resorting to a computer–generated design file (STL) [47,48,49]. Figure 7a briefly shows the process of manufacturing a dental prosthesis with AM technique. Similar to CAD/CAM technique, raw data is first acquired through intraoral scanning, followed by the building of 3D digital model with the aid of CAD software; second, an STL is constructed with the 3D digital model and transformed to 3D printing machine; third, each material layer is deposited one on top of the other within the 3D machine, consecutively, forming a three–dimensional part; fourth, some post–processing steps are needed to obtain the final dental prostheses, such as removal support, washing, and heat treatment [47].

Numerous AM techniques can be utilized to manufacture dental prostheses, including direct inkjet printing (DIP), selective laser melting (SLM), stereolithography (SLA), etc., [47,50]. DIP has been used by Özkol and his colleagues to prepare zirconia dental prostheses [51]. A tailored zirconia–based ceramic suspension was printed on a inkjet printer, followed by drying, debinding, and sintering. The sintered zirconia framework showed a relative density of >96% of the theoretical density and flexural strength of approximately 843 MPa [51] (Figure 7b). Gahler et al. combined layer–wise slurry deposition technique and SLM technique to prepare Al_2_O_3_–SiO_2_ dental ceramic components [52] (Figure 7c). After laser sintering, the density of Al_2_O_3_–SiO_2_ dental ceramic varied between 86% and 92% of the theoretical density. A thermal post–treatment (1600 °C in 2 h) is needed to enhance the density of the printed part (up to 90%) [52]. SLA has been applied by Hezhen et al. to fabricate the dental bridges and implants. A hybrid sol was prepared by mixing acrylates, methacrylates, 3Y–TZP powder, and photo initiator, followed by selectively curing of the photosensitive polymer in a printer. The printed part achieved geometries and dimensional accuracy, however, both macroscopic and microscopic defects were found after debinding and sintering, resulting in low strength [53].

Although the above AM technologies show great potentials in printing dental prostheses, there are some limitations to these techniques: 1. For DIP, high–quality inks or slurries are needed. Viscosity, surface tension, and ceramic powder/binder volume ratio need to be optimized to ensure the successful printing, which is a complicated and time–consuming process; 2. lack of high shape accuracy. As shown in Figure 7b,c, the printed parts prepared by DIP and SLM are lack of high shape accuracy to fulfil the requirements of dental prostheses; 3. post–thermal treatments are needed for the above three techniques. Obvious shrinkage occurs during the drying, debinding, and post–sintering process, which may lead to residual stress or even cracking in the sintered parts [53].

Compared to the work that has been done in the field of all–ceramic dental prostheses, glass–ceramic prepared with AM is, to some extent, a virgin land, especially in the field of dentistry. Darius and his colleagues have reported a method of manufacturing ZrO_2_–SiO_2_ glass–ceramic by combining ultrafast 3D laser nanolithography with calcination and sintering. Organic–inorganic hybrid sol–gel resin was first prepared, followed by SLM. The printed part can achieve a very high resolution of 100 nm. Post heat treatment enabled the formation of t–ZrO_2_ crystalline phase and inorganic amorphous SiO_2_ [54]. However, the authors did not state the application of the printed glass–ceramic. In the authors’ opinion, lots need to be done to fill the gap of dental glass–ceramic manufactured by AM.

## 6. Commercially Available and Newly–Developed Dental Glass–Ceramics

Since glass–ceramics started to be used in dentistry, materials with varied compositions have been developed. Table 1 lists three of the commercially available dental glass–ceramics, i.e., mica–based, leucite–based, lithium disilicate, and ZrO_2_–reinforced lithium silicate glass–ceramics. The physical properties of human enamel are also listed for comparison (Table 1). The following sections briefly introduce these dental glass–ceramics.

### 6.1. Mica–Based Dental Glass–Ceramic

**Figure 8:**
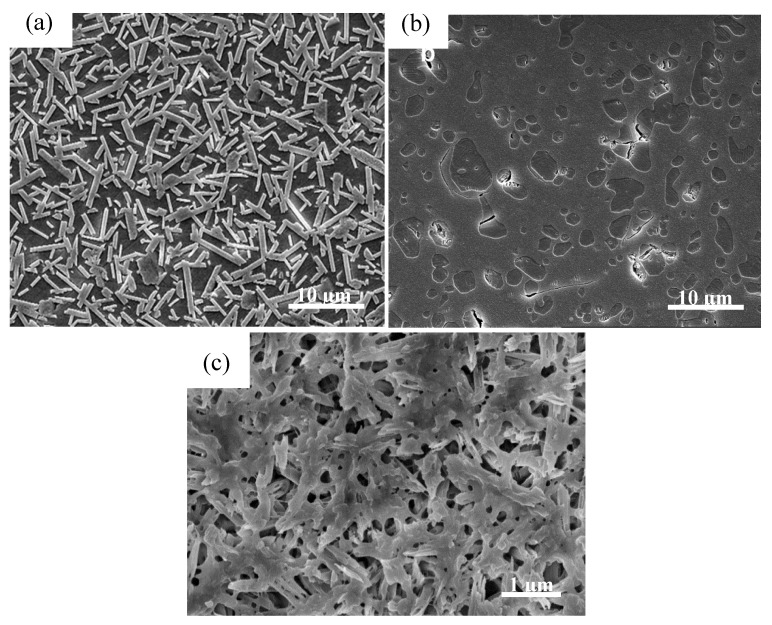
Typical microstructure of commercially available dental glass–ceramics (SEM images). (**a**) The mica glass–ceramic exhibited a typical “house of cards” microstructure with randomly interlocked mica platelets. Reprinted with permission of Ref [56]. (**b**) Leucite–based glass–ceramic (IPS Empress Esthetic) shows lamina–like, irregular–shaped leucite crystals. Reprinted with permission of Ref [58]. (**c**) Interlocked microstructure of IPS e.max Press. The glass phase has been removed by acid etching, leaving needle–like lithium disilicate crystals.

Mica–based glass–ceramics (SiO_2_–Al_2_O_3_–MgO–K_2_O–B_2_O_3_–F) are well-known glass–ceramics for dental restorations because of good machinability, bioactivity, and resemblance to tooth color. Mica–based glass–ceramics can be drilled and cut with conventional machining tools [61], thus, they can be easily manufactured to be of various geometries to fulfill different patients’ needs. The excellent machinability of mica–based glass–ceramics is attributed to their unique microstructure that consists of randomly interlocked mica platelets with a length of 2–5 µm, and a thickness of approximately 200 nm (Figure 8a). The randomly oriented plate– and lath–like crystals (Figure 8a) help in arresting fractures and deflecting cracks during milling and machining, which effectively prevents the cracks from propagating in a catastrophic manner [55,56]. Despite their recognized advantages, mica–based glass–ceramics show modest flexural strength (90–130 MPa, Table 1) and fracture toughness (0.8–1.5MPa·m^1/2^, Table 1). Thereby, in most cases, mica–based glass–ceramics are used as resin–bonded laminate veneers adhered to metal framework and posterior inlays [4]. Mica–based glass–ceramics are not strong enough to be used as all–ceramics dental prostheses, such as full anatomical crown and bridges.

### 6.2. Leucite–Based Dental Glass–Ceramic

Glass–ceramics based on leucite (KAlSi_2_O_6_) were developed as a leucite–containing porcelain composition that could be fired directly onto common dental alloys in 1962 [48]. The leucite crystalline show much higher CTE (~17 × 10^−6^ K^−1^) than that of a feldspar glass (~8 × 10^−6^ K^−1^) [4,57]. Porcelain frits with average CTEs (12−14 × 10^−6^ K^−1^) matching those of metallic framework can be produced by varying the proportions of leucite crystalline and feldspar glass [9]. A matching CTE between a porcelain veneer and metallic framework has two benefits: (1) Prevent the development of deleterious thermal stresses during manufacturing process; (2) avoid the chipping problem of porcelain fused to metallic framework in patients’ mouth. Compared to the glass matrix leucite crystals can be preferentially etched with acid, which allows leucite–based glass–ceramic to be utilized to create surface tomography features for resin bonding. This feature of leucite–based glass–ceramic makes the material very suitable for the veneering of metal frameworks [48]. In addition, a large amount of leucite crystalline (up to 35–50 wt%) can be incorporated into feldspar glass matrix without significantly compromising its translucency because the refractive index of leucite (*n* = 1.51) is very close to that of the feldspar glass (*n* = 1.52–1.53) [9]. This is beneficial to the improvement of mechanical properties. Meanwhile, leucite–based glass–ceramics offer the possibility of coloring the glass in natural tooth shades through the addition of metal oxide pigments. However, the strength of leucite–based glass–ceramics is still insufficient to be used as posterior fixed dental prosthetics (bridges). Leucite–based glass–ceramic is composed of lamina–like, irregular–shaped leucite crystals, with sizes ranging from 2–7 μm, as shown in Figure 8b. Typical commercial products made of leucite–based glass–ceramic are IPS Empress CAD and IPS Classic (Ivoclar Vivadent AG, Schaan, Liechtenstein) [15,62]. Their applications span from resin–bonded laminate veneers, to inlays and onlays, and to anterior and posterior crowns.

### 6.3. Lithium Disilicate (LD)

Currently, the most widely used and the strongest and toughest dental glass–ceramics are LD glass–ceramics. This class of glass–ceramic was commercialized for dental framework use and marketed under the trade name IPS Empress 2 in 1998 by Ivoclar Vivadent. However, IPS Empress 2 LD glass–ceramics had high clinical failure rates at 9% to 50% after 24 to 60 months [63], because of the insufficient flexural strength of this material for multiunit prostheses. Subsequently, a new and improved LD glass–ceramic (IPS e.max) with a much higher flexural strength (up to 400 MPa) was launched and the material gained popularity [14,15]. The IPS e.max LD glass–ceramics come in two forms, Press and CAD. IPS e.max Press is processed in the dental laboratory using the well–known lost–wax technique [14]. This technique is distinguished for providing high accuracy of fit.

As mentioned in 4.1, IPS e.max CAD was introduced in 2006 as an LD glass–ceramic, specifically prepared for CAD/CAM soft milling [15]. The material comes prepared in a “blue state,” which permits easier machining and intraoral occlusal adjustment [64]. In the “blue state,” the crystalline phase is lithium metasilicate (Li_2_SiO_3_) [65]. Once milling has been completed, the restoration is subjected to the second round of heat treatment, in which lithium metasilicate (Li_2_SiO_3_) reacts with the glass phase (SiO_2_) to form LD (Li_2_Si_2_O_5_), which is much stronger and tougher than the Li_2_SiO_3_. This is the so-called “soft milling,” that effectively reduce the wear of milling tool compared to “hard milling” (direct milling of sintered blocks). Figure 8c demonstrates the typical interlocked microstructure of LD glass–ceramic. The interlocked microstructure produces a high flexural strength that may reach up to 400 MPa and a fracture toughness up to 3 MPa·m^1/2^, which allows the use of LD as single restorations anywhere in the mouth and as short–span FDPs in the anterior region [14,44,66]. In addition to their good mechanical strengths, LD glass–ceramics also show advantages over other dental glass–ceramics in terms of adjustable shade and translucency. For instance, IPS e.max CAD is available in the standard A through D shades and also includes a line of bleach shades [44]. The color of the material can be adjusted by the content of the crystalline phase and also by colorant ions dispersed in the matrix. The primary ions consist of V^4+^/V^3+^ (blue/yellow), Ce^4+^ (yellow), and Mn^3+^ (brown) [15]. Besides, IPS e.max CAD also comes in three levels of translucency, medium opacity (MO), high translucency (HT), and low translucency (LT). This variation is accomplished via adjusting the crystal sizes with HT glass–ceramic exhibiting crystals of 1.5–0.8 mm, whereas LT glass–ceramic exhibits smaller crystals (0.8–0.2 mm) in a higher density [44].

### 6.4. Newly Developed Glass–Ceramic: ZrO_2_–SiO_2_ Nanocrystalline Glass–Ceramics

**Figure 9:**
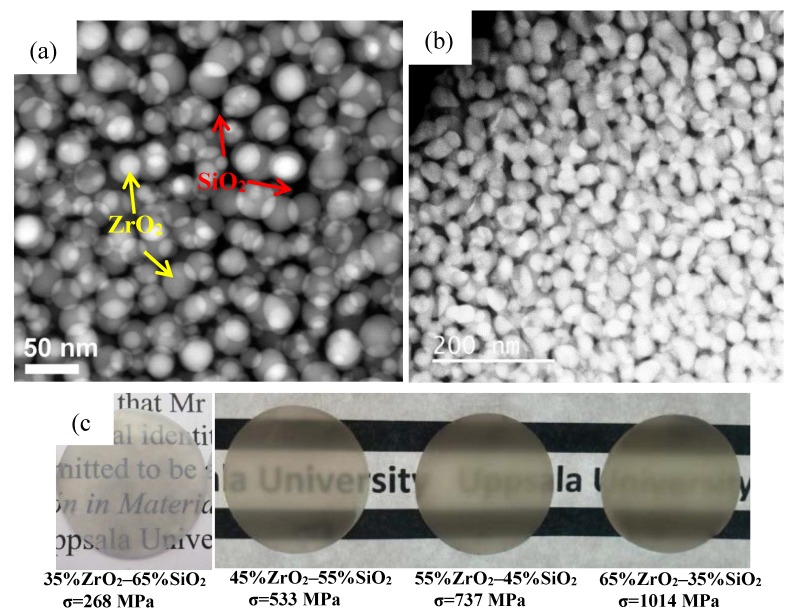
Scanning transmission electron microscopy (STEM) images of 35%ZrO_2_–65%SiO_2_ (mol%) (**a**) and 65%ZrO_2_–35%SiO_2_ (mol%) (**b**) glass–ceramics. As indicated in (a), nanoparticles with bright contrast are ZrO_2_ nanoparticles. Amorphous SiO_2_ matrix show dark contrast. Reprinted from Ref [33] and Ref [67]. (**c**) Optical images and the average flexural strength of the sintered ZrO_2_–SiO_2_ nanocrystalline glass–ceramics. The thickness of the glass–ceramics is approximately 1 mm. Reprinted from Ref [67].

Despite the great acceptance and broad use of LD glass–ceramics, the evolution of new dental materials has attempted to develop glass–ceramic with higher mechanical strength and higher translucency. In the last few years, we developed a strong and highly–translucent ZrO_2_–SiO_2_ nanocrystalline glass–ceramics [33,67]. In the glass–ceramics ZrO_2_ nanoparticles (NPs) are homogeneously embedded in an amorphous SiO_2_ matrix (Figure 9a,b). In the 35%ZrO_2_–65%SiO_2_ (mol%) glass–ceramic, ZrO_2_ NPs are spherical and isolated, and they have a size of approximately 20 nm (Figure 9a). In the 65%ZrO_2_–35%SiO_2_ (mol%) glass–ceramic, ZrO_2_ NPs become elliptic (Figure 9b). As can be seen from Figure 9c, the 35%ZrO_2_–65%SiO_2_ glass–ceramic is transparent. Although the translucency of the glass–ceramics slightly decreases with the increase of ZrO_2_ content, the 65%ZrO_2_–35%SiO_2_ glass–ceramic is still highly translucent (Figure 9c). The high translucency of ZrO_2_–SiO_2_ glass–ceramics makes the material an excellent candidate to be used as dental crown in terms of translucency. Strength is another important factor to be considered when developing a new dental glass–ceramic. The average flexural strength of 35%ZrO_2_–65%SiO_2_ glass–ceramic is 268 MPa [33]. The strength increases with the increase of ZrO_2_ content in the glass–ceramics, reaching as high as 1014 MPa for 65%ZrO_2_–35%SiO_2_ glass–ceramic [67]. The ZrO_2_–SiO_2_ glass–ceramics shows great potential to be used in dental restoration.

## 7. Strengthening Mechanisms of Glass–Ceramics

### 7.1. Interlocking Effect

**Figure 10:**
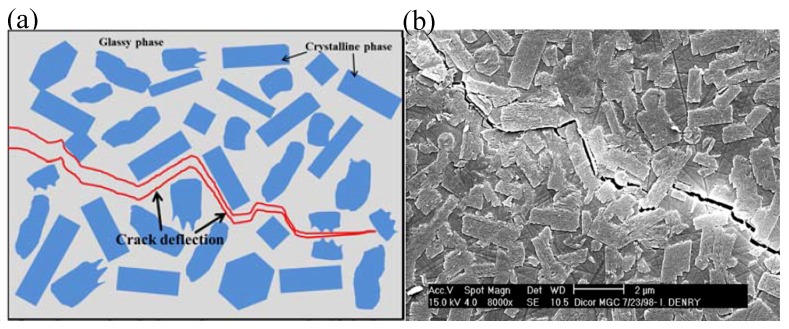
A schematic (**a**) and an example (**b**) demonstrating interlocking effect in glass–ceramics. The SEM image reveals crack deflection in a mica glass–ceramic. Reprinted with permission of Ref [56].

The basic microstructure of glass–ceramic is crystalline phases with certain shapes embedded in the glassy phase (Figure 3b). The predominate strengthening mechanism of glass–ceramic is correlated with “interlocking effect.” The peculiar crystals in the glass–ceramics formed interlocking microstructures, which could retard crack progression in the glass–ceramics, resulting in effective strengthening [59,68]. Figure 10a schematically demonstrate the strengthening mechanism, in which in most cases the crack propagates along with the crystals/glass phase interface, rather than traverse the dispersed crystals. In other words, intergranular fracture, rather than trans–granular fracture, is the main fracture mode in glass–ceramics [69,70]. Cracks propagate in a zig path (Figure 10a), instead of propagating in a direct path, which effectively consumes the energy of cracks [70]. An example of crack deflection caused by interlocking microstructures of mica glass–ceramic (Dicor MGC) is shown in Figure 10b.

### 7.2. ZrO_2_–Reinforced Glass–Ceramics

**Figure 11:**
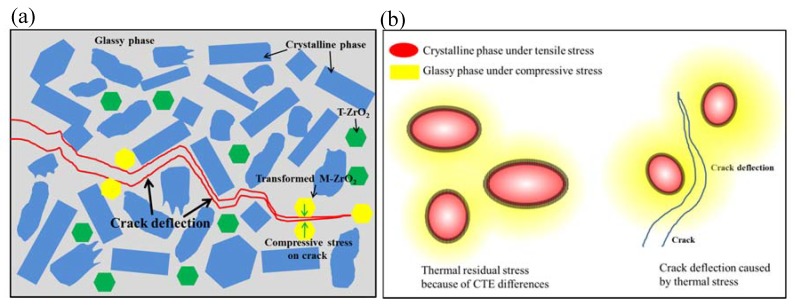
Schematics showing two traditional strengthening mechanisms of glass–ceramics. (**a**) Glass–ceramics can be strengthened by the addition of ZrO_2_ particles. Transformation toughening of ZrO_2_ particles contributes to the improvement of mechanical properties of glass–ceramics; (**b**) thermal residual stress arise in glass–ceramics upon cooling because of the thermal expansion mismatch between the crystalline and glass phases. The coefficient of thermal expansion (CTE) of the crystalline phase is larger than that of the glass phase. A propagating crack deviates from the crystalline phase, leading to crack deflection.

Incorporation of ZrO_2_ particles into the glass matrix is an effective method to improve the mechanical properties of glass–ceramics [60,68,71]. The strengthening mechanism of ZrO_2_ reinforced glass–ceramics is schematically demonstrated in Figure 11a. In addition to the crack deflection effect, the glass–ceramic is also strengthened by ZrO_2_ transformation toughening. The expansion in the volume of the ZrO_2_ grain accompanying the phase transition produces compressive stress on the crack, or micro–cracks around the crack, both consuming the energy of the main crack and thereby increasing the fracture resistance of the glass–ceramic [72,73].

A ZrO_2_ reinforced lithium silicate (Li_2_SiO_3_) glass–ceramic was introduced by Vita Zahnfabrick (Bad Säckingen, Germany) a few years ago. This new glass–ceramic is enriched with approximately 10 wt% ZrO_2_ particles. This newly developed generation of glass–ceramic combines the positive material characteristics of ZrO_2_ (high strength) and glass–ceramic (appealing aesthetics). Shaymaa et al. [60] found that ZrO_2_–reinforced lithium silicate glass–ceramic had significantly higher fracture toughness (2.31 ± 0.17 MPa m^1/2^) and flexural strength (444 ± 39 MPa) than those of ZrO_2_ free lithium disilicate glass–ceramic (IPS e.max CAD) with toughness and strength values of 2.01 ± 0.13 MPa m^1/2^ and 348 ± 29 MPa, respectively.

X. Huang et al. [74]. reported that the content of ZrO_2_ plays an important role in the strengthening effect of ZrO_2_ in lithium disilicate glass–ceramic. When the ZrO_2_ content was below 10 wt%, ZrO_2_ acted as nucleation agent and Li_2_Si_2_O_5_ crystals have a spherical morphology, instead of rod–like structure. This results in the loss of interlocking effect of Li_2_Si_2_O_5_ crystals and the decrease of mechanical strength. The addition of 15 wt% ZrO_2_ strengthens and toughens the glass–ceramic, with flexural strength increasing from 310 MPa (ZrO_2_ free) to 340 MPa (ZrO_2_ reinforced), and fracture toughness increasing from 2.2 MPa m^1/2^ to 3.5 MPa m^1/2^ [74]. Although the addition of ZrO_2_ particles improved the mechanical strength of lithium silicate glass–ceramic, the addition of ZrO_2_ particles causes some manufacturing problems. X.P. Chen et al. studied the machinability of ZrO_2_–reinforced lithium silicate, finding that of ZrO_2_–reinforced lithium silicate exhibited poorer machinability with high tangential and normal grinding forces and energy. Meanwhile, ZrO_2_ reinforced lithium silicate was the most difficult to machine compared with feldspar, leucite, and lithium disilicate glass ceramics. Edge chipping damage was found during milling. Thus, the manufacturing of high–strength ZrO_2_ reinforced lithium silicate is still a technical challenge [75].

Mica–base glass–ceramics can also be significantly reinforced by the addition of ZrO_2_ particles [55,73,76,77]. S. Galia et al. found that the mechanical strength of zirconia toughened mica glass–ceramics (containing 20 wt% YSZ) achieved a Vickers hardness of 9.2 GPa, an elastic modulus of 125 GPa, and an indentation toughness of 3.6 MPa·m^1/2^, which were close to those of lithium disilicate glass–ceramics (IPS emax.Press) [78]. At the same time, zirconia toughened mica glass–ceramic showed good cytocompatibility with human gingival fibroblast cells and better wear resistance with respect to commercial IPS emax Press. However, the zirconia toughened mica glass–ceramic had a coefficient of thermal expansion of 5 × 10^-6^/°C, which is lower than that of commercial core and veneering ceramics [79].

### 7.3. Thermal Residual Stress

Thermal residual stresses, arising in glass–ceramics upon cooling, plays an important role in the mechanical properties of glass–ceramics. These stresses are raised from the thermal expansion and the elastic mismatch between the crystalline and glass phases. The mechanical properties of glass–ceramics are depended not only on their composition and microstructure but also on the type (tension or compression) and the magnitude of these residual stresses [80]. Figure 11b schematically demonstrates the formation of thermal residual stress and its effect on crack propagation. According to the model proposed by Selsing [80], the coefficient of thermal expansion (CTE) of the crystalline phase is larger than that of the glass phase. The thermal residual stress field in the glass–ceramic consists of two regions: the tensile stress region (represented by red color) in the crystalline phase and the compressive region (represented by yellow color) in the matrix [67,81], as schematically shown in Figure 11b. A propagating crack will deviate from the crystalline phase, resulting in crack deflection and fracture resistance improvement (Figure 11b).

Thermal residual stresses effect has been thought to be a key factor for strengthening leucite glass–ceramics. The CTE of the tetragonal leucite phase was much higher than that of glass matrix [57,58], which results in residual tensile stresses inside tetragonal leucite crystals along the radial direction at room temperature [82]. This tensile stress would be balanced by residual compressive stresses in the glass matrix along the tangential direction [82]. According to the schematic (Figure 11b), the residual stress states in leucite glass–ceramics is beneficial to improve their strength and toughness. In fact, thermal residual stresses also exist in lithium disilicate (LD) glass–ceramics since the CTE of orthorhombic LD phase and the glass matrix were estimated to be 10.1−10.8 × 10^−6^/K and 12.2−12.8 × 10^−6^/K [80,82], respectively. Thus, the residual stress in LD glass–ceramics is opposite to that in leucite glass–ceramics, being compressive stresses inside the crystals and tensile stresses in the glass matrix. The effects of this residual stress states on the mechanical behavior of LD glass–ceramics remains unclear. It was thought that the residual stresses had a negligible effect on the mechanical properties of LD glass–ceramics since LD glass–ceramics have strong “interlocking effect” because of the rod–like LD crystals [16].

### 7.4. Strengthening by 3D Nanoarchitecture

**Figure 12:**
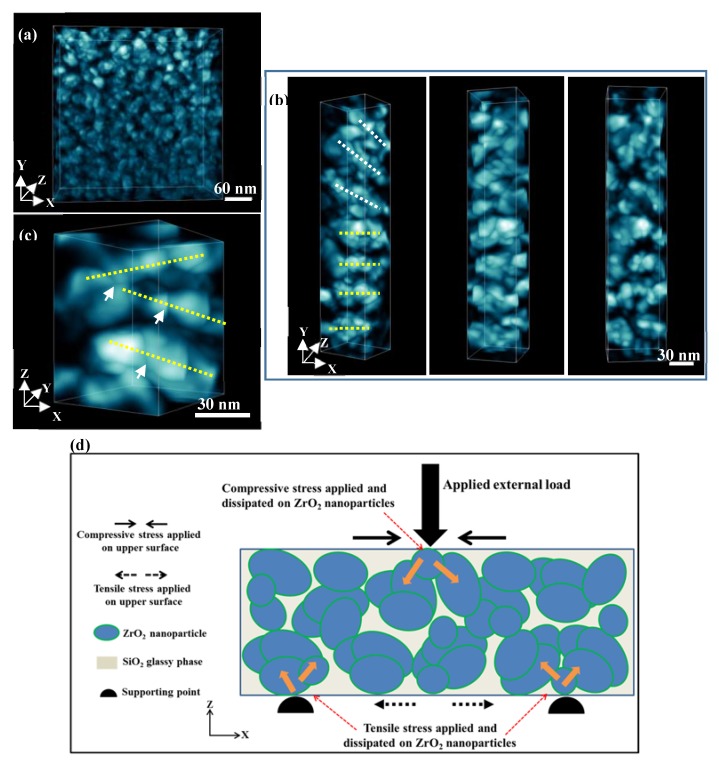
A new strengthening mechanism of glass–ceramic. (**a**–**c**) 3D nanoarchitecture built by ZrO_2_ NPs in 65%ZrO_2_–35%SiO_2_ (mol%) glass–ceramic revealed by electron tomography. Reprinted from Ref [83]. (**d**) A front view schematic diagram of stress state of the glass–ceramic during the bending test.

As mentioned in Section 6, we developed a new ZrO_2_–SiO_2_ nanocrystalline glass–ceramic system, among which 65%ZrO_2_–35%SiO_2_ (mol%) glass–ceramic achieved a flexural strength of as high as 1014 MPa [67]. To address the underlying strengthening mechanism, we characterized the 65%ZrO_2_–35%SiO_2_ (mol%) glass–ceramic with STEM [83]. The results are demonstrated in Figure 12a–c, in which the colored contrast corresponds to ZrO_2_ NPs. Figure 12a gives an overview of the 3D spatial distribution of ZrO_2_ NPs. Three long columns (Figure 12b) were extracted from the overview image, showing the formation of short nanofibers. The ZrO_2_ nanofibers are composed of ZrO_2_ NPs connected by grain boundaries (marked by white arrows, Figure 12c). The ZrO_2_ NPs in the glass–ceramic form 3D nanoarchitecture. In a composite material, the crystalline phase and the matrix may have different Young’s modulus values. When the crystalline phase and the matrix are deformed to the same strain, the two components bear different stresses because of the difference in Young’s modulus values [84]. If the crystalline phase is much stiffer (with higher Young’s modulus value) than the matrix, then the crystalline phase carries more load than the matrix [84]. In the ZrO_2_–SiO_2_ glass–ceramic, the tetragonal ZrO_2_ NPs (~ 210 GPa [85]) are much stiffer than amorphous SiO_2_ (~ 70 GPa [86]). Thus, the external load is applied and dissipated on ZrO_2_ NPs when the material was loaded (Figure 12d). It is the ZrO_2_ 3D nanoarchitecture that carries the majority of the external load, rather than the SiO_2_ matrix, which greatly contributes to the high strength of the 65%ZrO_2_–35%SiO_2_ (mol%) glass–ceramic [83]. The new strengthening mechanism of the glass–ceramic offer us an idea of designing and manufacturing high–strength glass–ceramics, i.e., to form 3D nanoarchitecture built by stiff nanoparticles.

## 8. Future Glass–Ceramics in Dentistry

**Figure 13:**
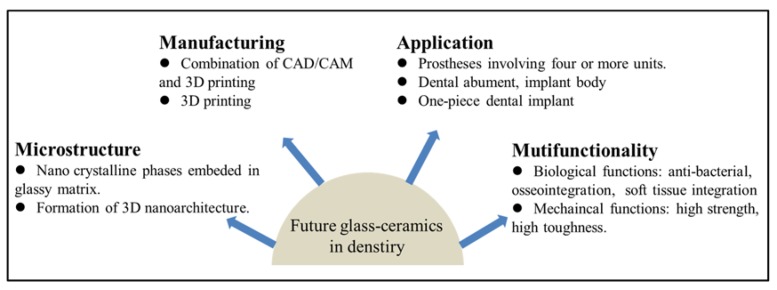
A perspective and outlook for future directions in developing new dental glass–ceramics.

Glass–ceramics have been used as dental prostheses since the 1980s. Great process in improving the properties of dental glass–ceramics has been achieved in the past decades. Here, we provide some future directions in developing new dental glass–ceramics, which may offer inspiration to the dental community (Figure 13).

Microstructure: As can be seen from Figure 8, the sizes of the crystalline phase in the commercially available dental glass–ceramics are in the micrometer scale [55,65,82]. If we can reduce the size of the crystalline phase to the nanometer scale, the glass–ceramics may show some unexpected properties. For instance, the length of rod–like Li_2_Si_2_O_5_ crystals in LD glass–ceramic is 3–6 µm. If the length of Li_2_Si_2_O_5_ crystals can be reduced to nanometer range with a certain method, then the crystals become needle–like ones. The LD glass–ceramic may show much higher strength and toughness, as well as high translucency. Moreover, if the nano–sized crystalline phase(s) can form 3D interconnected nanoarchitecture, the glass–ceramics may show ultrahigh strength. One successful example is the ZrO_2_–SiO_2_ nanocrystalline glass–ceramics (Figure 9).

### 8.1. Manufacturing

As discussed in 5.1, there exist two major drawbacks of CAD–CAM: wearing of milling tool and a great waste of dental material. Lots can be done in manufacturing dental glass–ceramics with AM technique. The combination of CAD/CAM and 3D printing would be an interesting attempt in the near future. This is called hybrid manufacturing (HM) [87]. Raw dental prostheses are first manufactured with 3D printing, followed by CAD/CAM to obtain the final dental prostheses. The hybrid manufacturing (HM) combines the advantages of additive manufacturing, such as few limits in shape reproduction and minimization of waste materials, with the advantages of subtractive manufacturing, such as high accuracy of dimensional tolerances [87].

### 8.2. Application

Currently, dental glass–ceramics are mainly used as dental crown. Stronger and tougher glass–ceramics need to be developed to broaden their applications, not only for dental crown, but also for the dental abutment, implant body, or even one–piece implant. The dental implant made by glass–ceramics would effectively avoid the issues of a dental implant made by titanium or titanium alloys, such as titanium allergy, accumulation of metal ions, and poor aesthetic.

### 8.3. Multifunctionality

Dental implant can be possibly made by glass–ceramics in the future. Meanwhile, the existence of a glassy matrix in glass–ceramic offers great potential to functionalize the material by incorporating ions into the matrix [88]. For instance, bioactive ions, such as Mg^2+^, Ca^2+^, Sr^2+^, can be easily incorporated into the glassy matrix. Once the dental implant made by glass–ceramics is implanted in the human body. The bioactive ions can release from the implant surface to improve the osseointegration and soft tissue integration of the implant in the early stage. The mechanism is similar to bioglass. Moreover, ions that have anti–bacterial property, such as Ag^+^ or Cu^2+^, may also be incorporated into the glassy matrix to introduce the antimicrobial activity to the implant. Thus, the glass–ceramic dental implant not only provides mechanical support to dental restoration but also shows excellent biological properties.

## 9. Summary

In dentistry, glass–ceramics with appealing aesthetics and adequate strength are attracting extensive attention of material scientists, dentists, and patients. Mica–based, leucite–based, and lithium disilicate glass–ceramics are the most prevalent clinically used examples of dental glass–ceramics. However, compared with sintered dental ceramics (e.g., Y–TZP), commercially available dental glass–ceramics have much lower strength and fracture toughness that limits their wider applications in dentistry, such as multi–unit prostheses involving molar restoration, thereby improving the strength and toughness of glass–ceramics, meanwhile maintaining their high translucency is the main task of optimizing the existing or developing new glass–ceramics. As for the manufacturing method, additive manufacturing shows great potential. However, a lot of issues, such as low accuracy, need to be solved before additive manufacturing becomes the predominate manufacturing in dentistry. Glass phase can be modified to incorporate with specific ions, e.g., Ag^+^, Cu^2+^, and Sr^2+^, to simultaneously introduce more than one desirable biological properties to glass–ceramics.

## Figures and Tables

**Table 1:** Part of commercially available glass–ceramics and their microstructure, physical properties, and clinic indication. σ, Hv, KIc, E, CTE represent flexural strength, Vickers hardness, fracture toughness, elastic modulus, and coefficient of thermal expansion.

Glass–Ceramic	Commercial Brand	Crystalline Microstructure	Manufacturing Technique	Mechanical Properties & CTE	Clinic Indication
**Mica–based [4,55,56]**	Dicor^®^ (Corning Inc, Dentsply), Cera Pearl^®^ (Kyocera Corp)	Morphology: plate–like crystals; Composition: K_2_Mg_5_Si_8_O_20_F_4_; Size: 2–5 µm (length), ~200 nm (thickness)	Castable CAD/CAM	σ: 90–130 MPa Hv: 4–6.5 GPa KIc: 0.8–1.5 MPa·m^1/2^ E: ~70 GPa CTE:6.4–7.2 × 10^−6^ K^−1^	Resin–bonded laminate veneers, anterior crowns, posterior inlays
**Leucite–based [4,57,58]**	IPS Empress^®^, IPS Empress^®^ CAD (Ivoclar), Optimum Pressable CeramicOPC^®^ (Jeneric/Pentron), Finesse^®^ (Dentsply)	Morphology: lamina–like crystals (35–50 wt%); Composition: tetragonal KAlSi_2_O_6_; Size: 1–4 µm	Hot press CAD/CAM	σ: 80–120 MPa Hv: ~6.5 GPa KIc: 0.7–1.2 MPa·m^1/2^ E: ~70 GPa CTE:16.6 × 10^−6^ K^−1^ (100–400 °C), 17.5 × 10^−6^ K^−1^ (100–500 °C)	Resin–bonded laminate veneers, inlays, onlays, and crown
**Lithium disilicate [4,27,29,59]**	IPS e.max Press^®^, IPS e.max CAD^®^ (Ivoclar)	Morphology: needle–like crystals (approx. 70 vol%); Composition: Li_2_Si_2_O_5_; Size: 3–6 µm (length)	Hot press CAD/CAM	σ: 350–450MPa Hv: 4–6.5 GPa KIc: 0.8–1.5MPa·m^1/2^ E: ~70 GPa CTE:10.2 ± 0.4 × 10^−6^K^−1^ (100–400 °C), 10.6 ± 0.35 10^−6^ K^−1^ (100–500 °C)	Resin–bonded laminate veneers, inlays and onlays, crowns, bridges in the anterior region up to premolars
**Zirconia reinforced lithium silicate [60]**	Vita Suprinity^®^ (Vita Zahnfabrick, Bad Säckingen, Germany)	Morphology: homogeneous fine Li_2_SiO_3_ crystals, ZrO_2_ particles (~70 wt%);	CAD/CAM	σ: 444 ± 39 MPa Hv: 6.5 ± 0.5 GPa KIc: 2.31 ± 0.17 MPa·m^1/2^ E: 70 ± 2 GPa	inlays, onlays, veneers, anterior and posterior crowns, single tooth restorations on implant abutments
**Enamel [12]**	–	Hydroxyapatite crystals (approx.90 vol%); Composition: Ca_5_(PO_4_)_3_OH; Size: 3–6 µm (length)	–	σ: 260–280 MPa Hv: 3–5 GPa KIc: 0.6–1.5 MPa·m^1/2^ E: 70–100 GPa	–
